# Enhanced analysis of tabular data through Multi-representation DeepInsight

**DOI:** 10.1038/s41598-024-63630-7

**Published:** 2024-06-04

**Authors:** Alok Sharma, Yosvany López, Shangru Jia, Artem Lysenko, Keith A. Boroevich, Tatsuhiko Tsunoda

**Affiliations:** 1 Laboratory for Medical Science Mathematics, RIKEN Center for Integrative Medical Sciences, Yokohama, Japan; 2 Institute for Integrated and Intelligent Systems, Griffith University, Brisbane, Australia; 3 Laboratory for Medical Science Mathematics, Department of Biological Sciences, School of Science, The University of Tokyo, Tokyo, Japan; 4 Azenta Life Sciences, Tokyo, Japan; 5 Laboratory for Medical Science Mathematics, Department of Computational Biology and Medical Sciences, Graduate School of Frontier Sciences, The University of Tokyo, Tokyo, Japan

**Keywords:** Computational biology and bioinformatics, Mathematics and computing

## Abstract

Tabular data analysis is a critical task in various domains, enabling us to uncover valuable insights from structured datasets. While traditional machine learning methods can be used for feature engineering and dimensionality reduction, they often struggle to capture the intricate relationships and dependencies within real-world datasets. In this paper, we present Multi-representation DeepInsight (MRep-DeepInsight), a novel extension of the DeepInsight method designed to enhance the analysis of tabular data. By generating multiple representations of samples using diverse feature extraction techniques, our approach is able to capture a broader range of features and reveal deeper insights. We demonstrate the effectiveness of MRep-DeepInsight on single-cell datasets, Alzheimer's data, and artificial data, showcasing an improved accuracy over the original DeepInsight approach and machine learning methods like random forest, XGBoost, LightGBM, FT-Transformer and L2-regularized logistic regression. Our results highlight the value of incorporating multiple representations for robust and accurate tabular data analysis. By leveraging the power of diverse representations, MRep-DeepInsight offers a promising new avenue for advancing decision-making and scientific discovery across a wide range of fields.

## Introduction

Tabular data analysis plays a crucial role in various fields, ranging from biomedical research to finance and beyond. The ability to extract meaningful insights from structured datasets is vital for making informed decisions and has a potential to drive advancements in diverse domains. Traditional machine learning (ML) methods, such as feature engineering and dimensionality reduction, have long been employed to uncover patterns and relationships within tabular data^1–5^. However, these approaches often struggle to capture the complex interactions and dependencies inherent in real-world datasets^6–8^.

The fundamental approach for ML methods involves processing a column vector of size $d \times 1$ to extract pertinent information essential for classification or regression. However, as the complexity of tabular data continues to grow, ML techniques face challenges in identifying relevant class types. For instance, accurately determining phenotypes through the processes of feature extraction and classification plays an increasingly important role in diagnosis and disease analysis. It is important to note that feature selection has wide utility across various research domains, extending beyond genomic data analysis. Hence, the procedures of feature selection, feature extraction, and classification significantly impact the reliability of ML algorithms. However, traditional ML approaches disregard crucial neighborhood information and treat each component of a sample as independent of the others.

On the contrary, two-dimensional convolutional neural networks (CNNs) are one of the deep learning architectures that have shown immense potential in the field of image analysis^9^. What sets CNNs apart from traditional ML methods is their unique approach to feature extraction and classification, accomplished using convolutional layers that process an input image structured as a $p \times q$ feature matrix. The advantages of CNNs include efficient feature extraction from spatially coherent pixels and the ability to facilitate deeper networks with fewer parameters through weight sharing^10^, detection of higher-order relationships and nonlinear correlations, and the ability to achieve remarkable performance even with a reduced number of samples.

A key strength of CNNs lies in their adeptness at harnessing the spatial relationships intrinsic to images. By effectively extracting features from adjacent pixels, CNNs capitalize on the wealth of information shared by nearby pixels. This enables them to capture intricate spatial patterns that traditional ML approaches often overlook. As a result, CNNs present a highly effective solution for tasks where spatial context plays a crucial role in the accurate analysis and classification of data.

Building upon this concept, DeepInsight^6^, introduced a data conversion methodology. It transforms a non-image feature or column vector from a tabular dataset $\chi \in \Re^{d}$ into an image. A total of $n$ samples, $x \in \chi$ (feature vectors) of size $d \times 1$ each are converted to $n$ images of size $p \times q$ each. In this methodology, $\chi$ is first transposed to create a feature set $G \in \Re^{n}$. This set, $G$, undergoes some manifold technique like t-distributed stochastic neighbor embedding (t-SNE)^11^ or Uniform Manifold Approximation and Projection (UMAP)^12^, to obtain a 2D Cartesian plane, where points represent feature locations, not the feature itself. Then, a convex-hull algorithm identifies the smallest rectangle containing all the points. This is then aligned horizontally or vertically via rotation. Finally, Cartesian coordinates are converted into pixels, and feature values are mapped to these pixel locations, creating an image representation. This process establishes spatial relationships between features, enhancing the feature extraction capabilities. Recent advancements have extended the utility of DeepInsight beyond CNN architectures and classification tasks^7,8,13–18^.

Also, to enhance the capabilities of the DeepInsight suite, we leverage the power of CNNs pre-trained with large datasets of images and utilize transfer learning. Transfer learning allows us to benefit from the knowledge learned by CNNs from extensive image datasets, such as ImageNet, without training the entire network from scratch. By initializing the CNNs with these pre-trained weights, the model gains the ability to extract high-level and spatial features effectively, even when applied to tabular data represented as images. This integration of transfer learning significantly enhances the feature extraction process within the DeepInsight framework, enabling more robust and accurate analysis of complex tabular datasets.

With the advent of techniques like DeepInsight^6^, the repertoire of deep learning techniques for tabular data analysis has expanded, offering the promise of automated feature learning and improved predictive accuracy^6,7,13–20,20,21^. DeepInsight has consistently demonstrated success in uncovering hidden patterns for various kinds of data^13,14,18^ and was used as part of the winning model in the Kaggle.com competition hosted by MIT and Harvard University^22^. Nevertheless, there remains room for further improvements, as currently DeepInsight primarily operates on a single representation of the data.

In this paper, we propose an extension of DeepInsight, termed Multi-representation DeepInsight or MRep-DeepInsight, which extends the DeepInsight framework through the integration of multiple manifold techniques. This development addresses a key limitation of the original method: the fixed positioning of data elements. In CNNs, data augmentation via rotation, translation, reflection or random scaling is often used for mitigating issues such as overfitting and data scarcity, thereby improving model robustness and generalization. However, within the DeepInsight framework, the fixed relationship between two data elements imposes constraints on such augmentation possibilities (although averaging of two transformed image samples is feasible). This fixed positioning can adversely impact the model’s robustness, especially in cases of limited training data. MRep-DeepInsight addresses this challenge by introducing greater flexibility. By introducing the multiple manifold model, MRep-DeepInsight enhances the robustness and flexibility of feature extraction from structured data. It allows for the dynamic positioning of each element within the feature vector, denoted as $L\left( g \right)$, across multiple manifolds. This approach enables an element, $g$, to assume multiple positions, represented as $L(g|\lambda_{r} )$, where $\lambda_{r}$, is the $r$-th manifold technique, for $r = 1,2,...,m$. This approach, which integrates multiple manifold techniques, is analogous to an ensemble of kernels, each providing a unique perspective on the data. In MRep-DeepInsight, we integrate these perspectives as $\cup_{r = 1}^{m} L(g|\lambda_{r} )$, in contrast to the singular $L\left( g \right)$ positioning in the original DeepInsight framework. This multiplicity in positioning not only enhances the capability of the model to represent and process data more effectively but also contributes to a more robust learning process.

**Figure 1:**
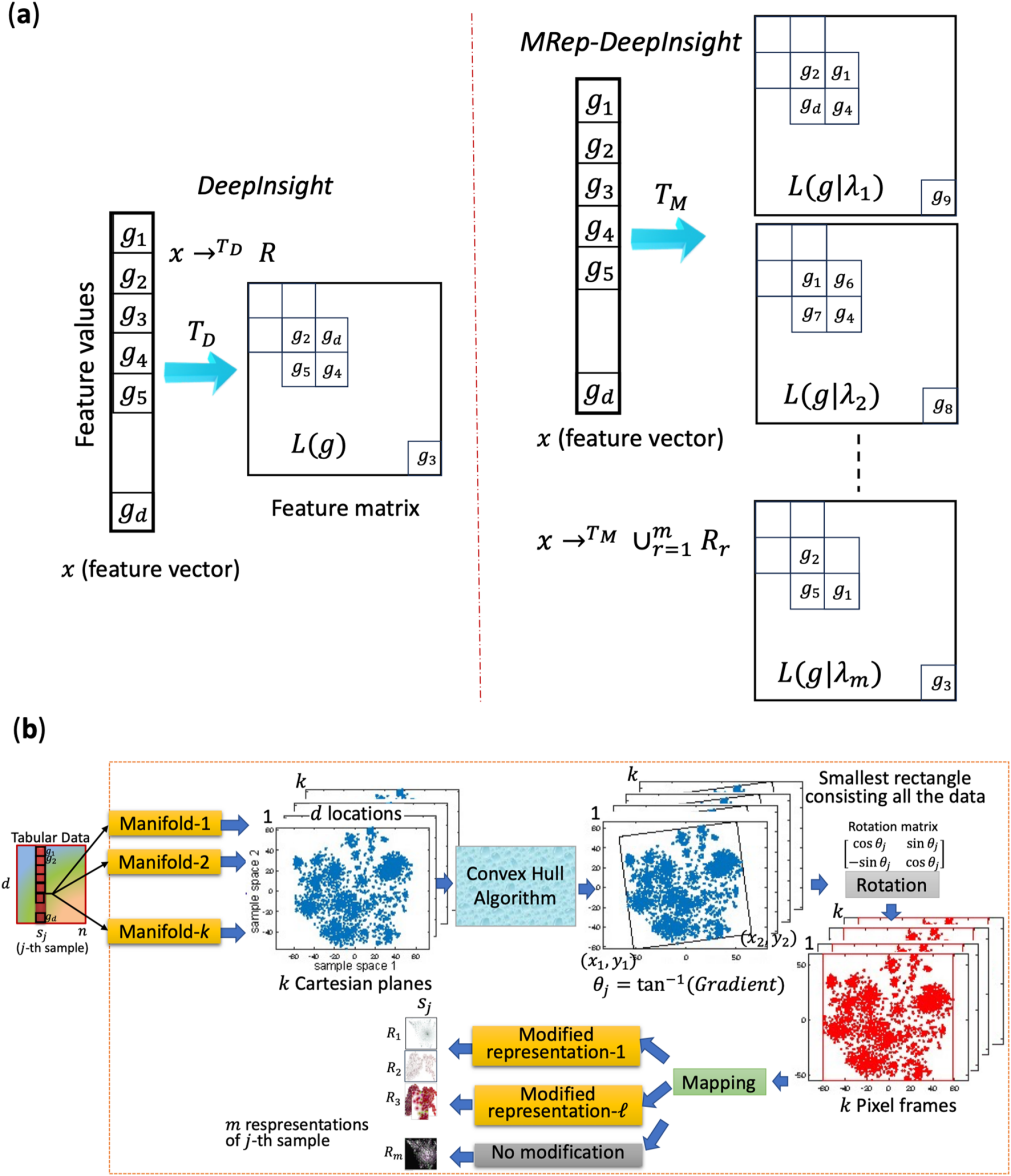
Schematic overview of the MRep-DeepInsight method. (**a**) Demonstrates the transformation difference between the original DeepInsight and MRep-DeepInsight, showcasing the shift from a single representation to multiple representation for each feature vector. (**b**) Depicts the MRep-DeepInsight pipeline, illustrating the steps involved in converting tabular data into a set of image samples with varied representations, from manifold to the final image conversion.

The core motivation behind MRep-DeepInsight is to leverage the complementary nature of different data representations. By simultaneously employing various established feature mapping techniques, such as UMAP^12^, t-SNE^11^, blurring technique^23^, and Gabor filtering^24^, we generate multiple representations of each sample, thereby providing a comprehensive view of its underlying characteristics. Theoretically, with an infinite number of manifold techniques, we could represent any nonlinear function, reiterating the principle in neural networks where an infinite number of nodes in middle layers allows for the representation of any function. However, given practical constraints such as limited data sizes and fixed hardware capacities, utilizing a set of established feature mapping techniques can achieve better performance. Therefore, we have selected these specific techniques for their demonstrated effectiveness in various contexts. Nonetheless, MRep-DeepInsight offers flexibility, allowing users to choose their preferred mapping techniques relevant to their domain. The framework is designed to integrate any number of techniques, limited only by hardware capabilities. These models act similarly to kernels in machine learning, extracting distinct and valuable features from the data. These features are then synergistically integrated within the MRep-DeepInsight framework, enabling more robust and accurate analysis of tabular data. An overview depicting the MRep-DeepInsight method for classification tasks is shown in Fig. 1 (see Methods for details). The difference between the transformation of DeepInsight and MRep-DeepInsight is depicted in Fig. 1a. The MRep-DeepInsight pipeline is shown in Fig. 1b. Furthermore, a detailed comparison of the representations produced by DeepInsight and MRep-DeepInsight is illustrated in Supplement Figure S4, accompanied by a discussion that highlights the distinctions between the two methodologies.

The DeepInsight framework, along with its subsequent enhancements, primarily focuses on converting data from tabular to image format. Initially employed for classification tasks using various CNN architectures, ranging from ResNet to the more advanced Noisy-Student EfficientNets^22,25^, as well as custom-made networks. The framework is also compatible with Vision Transformers (ViT)^26^. The applications of DeepInsight, however, extend beyond just classification tasks. It has been effectively used for feature selection^27,28^, integrated with the methods like XGBoost and random forest^29,30^, identifying gene subsets for pathway analysis^8^, generating data through GANs^30,31^, and processing multi-omics data via multiple layers^7^. This wide range of applications highlights the potential of MRep-DeepInsight as a generalized tool for diverse data analysis and interpretation.

In this paper, we demonstrate the improvement of the DeepInsight framework through an integrated model to enrich data characterization. This improvement is demonstrated by measurably better classification accuracy. While MRep-DeepInsight was used in combination with a ResNet classifier, the design is not limited to this architecture. The choice of ResNet in this study serves to highlight the improvements brought about by MRep-DeepInsight over the DeepInsight framework when applied within the same CNN architecture.

This adaptability of DeepInsight is further evidenced by its compatibility with various models, including Vision Transformers (ViTs) and XGBoost. This expansion of the framework signifies its evolution from a tool focused on specific tasks to a more comprehensive instrument for data representation and analysis. DeepInsight, therefore, offers a multifaceted approach to data interpretation, enabling deeper insights across various applications.

In this paper, we have shown the utility of MRep-DeepInsight on single-cell datasets, Alzheimer's data, and artificial datasets. By incorporating multiple representations, we aim to capture intricate relationships, interactions, and nonlinearities often overlooked by traditional ML methods or even the original DeepInsight approach.

In this paper, we present a thorough evaluation of MRep-DeepInsight on a diverse set of tabular datasets. The presented results demonstrate the effectiveness of the MRep-DeepInsight approach in improving classification accuracy by uncovering important latent patterns. We also discuss the implications of MRep-DeepInsight for various domains that rely on accurate and comprehensive tabular data analysis.

The detailed technical aspects of these theoretical advancements, and their implications for the functionality and performance of the MRep-DeepInsight model, are further elaborated in the Methods section of this paper. Overall, MRep-DeepInsight method contributes to the field of tabular data analysis, offering a novel and effective approach for extracting insights from structured datasets.

## Results

### Experimental setup

To evaluate the effectiveness of the MRep-DeepInsight method, we conducted experiments using six diverse datasets and compared the results with state-of-the-art classifiers. The dataset collection consists of the following: 1 single-cell dataset containing scRNA-seq and ATAC-seq data, 1 single-cell dataset with scRNA-seq only, 1 single-cell dataset with ATAC-seq only, 1 miRNA expression dataset of Alzheimer disease, and 2 artificial datasets (Ringnorm and Madelon). Our primary objective is to demonstrate improved classification accuracy by converting non-image data into multiple representations and processing them using the CNN architecture implemented in the MRep-DeepInsight method. In this study, we utilized two nets, ResNet-50^32^ and EfficientNet-B6^33^. In selecting ResNet-50 and EfficientNet-B6 for our study, we aimed to build upon prior related work while exploring the cutting-edge in neural network architecture. ResNet-50 was chosen due to its proven effectiveness and use in our previous study^8^, ensuring continuity and comparability. EfficientNet-B6, recognized for its efficiency in the Kaggle.com competition^22^.

The datasets were divided into training, validation, and test sets in an 80:10:10 proportion, respectively. This split is a commonly used standard as evidenced by studies like Kim et al.^34^, Ray et al.^35^, Struble et al.^36^, and Xu and Zhang^37^. Due to intensive computational resources required for training deep learning models, we followed a practical approach similar to recent studies in the field^25,33,38^. The model was trained on the training set, and its fitness was evaluated using the validation set. Hyperparameters were selected to minimize the validation error, particularly for the ResNet-50 architecture. Default hyperparameters were mostly used for the EfficientNet-B6 architecture. Importantly, the test set was completely separated from the training and model fitting stages to ensure an unbiased assessment of the final model's performance. Classification accuracy, defined as the percentage of correctly classified samples from the test set, was computed as the evaluation metric.

The description of the datasets used in our experiments is as follows: The single-cell data is sourced from PBMC reference, containing scRNA-seq or gene expression profiles with 9602 samples, where scRNAseq consists of 378 genes or dimensions. Additionally, the dataset contains ATAC-seq profiles with corresponding samples and 578 dimensions. This dataset has been obtained from the 10 × Chromium Next GEM Single Cell Multiome ATAC + Gene Expression sequencing platform and analyzed using the Cell Ranger ARC pipeline, which includes counting UMIs based on the fastq data .

Notably, batch normalization was not performed on this dataset to preserve the original features. It constitutes 17 distinct cell types, posing a multi-class classification challenge. Among the majority of cell types are CD14 monocyte, CD4 naive, CD8 naive and CD4 TCM (see Supplement Table S4 for the full list of cell types). Notably, CD14 monocyte stands as the predominant cell type in the dataset, accounting for over 26.5% of the samples (same as previously reported^39^). Furthermore, we utilized the Alzheimer's disease (AD) dataset from a previous study^40^, which comprises 1309 samples of AD and normal controls (NC); details of AD dataset can be seen in Supplement Table S5. Lastly, we incorporated two artificial datasets. The first one is Madelon^41^, which consists of 2600 samples and 500 dimensions. It represents a two-class classification problem with continuous input variables, and it exhibits multivariate and highly non-linear characteristics. The second artificial dataset is ringnorm^42^, which is a 20-dimensional, two-class classification problem with 7400 samples. Each class is drawn from a multivariate normal distribution, where class 1 has a zero mean and four times the identity covariance, while class 2 has a mean of $2/\sqrt {20}$ with unit covariance. More information on the artificial datasets is given in Supplement Table S6. A summary of these datasets can be found in Table 1.

**Table 1:** Summary of datasets used.

Datasets	#Samples	#Features	#Classes	Types
Single-cell	9602	1912	17	scRNA-seq + ATAC-seq
Single-cell	9602	956	17	scRNA-seq
Single-cell	9602	956	17	ATAC-seq
Alzheimer	1309	2539	2	miRNA
Madelon	2600	500	2	Artificial
Ringnorm	7400	20	2	Artificial

### Performance comparison

In this section, we compare the performance of MRep-DeepInsight with existing state-of-the-art classifiers, such as random forest, L2-regularized logistic regression, (details in Sect. 7 of Supplement File 1), XGBoost^43^, LightGBM^44^ and FT-Transformer^45^, and DeepInsight. For all competing methods (random forest, logistic regression, XGBoost, LightGBM, and FT-Transformer), the dataset was segmented into three distinct parts: a training set, a validation set and a test set, distributed in the ratios of approximately 80%, 10%, and 10%, respectively. The training set was used for initial training of the model, the validation set for fine-tuning and parameter optimization, and the test set to assess the performance of the fully optimized model. Details on the parameters used for optimization of competing models are discussed in Sect. 10 of Supplement File 1. The image transformation parameters of DeepInsight are outlined in Sect. 11 (Supplement Table S15), noting that default values were used for image transformation while CNN hyperparameters were optimized using Bayesian optimization.

The purpose of this comparison is to demonstrate how MRep-DeepInsight enhances the characteristics of the data by integrating multiple mapping schemes, leading to improved classification performance. We evaluated the performance on six diverse datasets, and the classification accuracy results are summarized in Table 2 (refer to Supplement Tables S2, S3 for a brief discussion on hyperparameters).

**Table 2:** Classification accuracy (in percentage) comparison across datasets and models.

Datasets	Random forest	Logistic regression	XGBoost	LightGBM	FT-transformer	DeepInsight *(baseline)*	MRep-DeepInsight (ResNet50)	Representations used for MRep-DeepInsight
Single-cell: scRNA-seq + ATAC-seq	83.3	88.9	79.0	75.3	88.7	88.2	**90.5**	*tsne (hamming)* + *umap* + *Blurring*
Single-cell: scRNA-seq	84.6	87.7	84.4	73.4	86.2	89.2	**90.0**	*tsne (hamming)* + *tsne (Euclidean)* + *umap* + *Gabor*
Single-cell: ATAC-seq	70.9	79.0	65.7	61.2	76.3	78.0	**79.4**	*tsne (hamming)* + *tsne (Euclidean)* + *umap* + *Gabor*
Alzheimer	81.7	87.0	77.9	77.9	**90.1**	87.0	89.3	*tsne (hamming)* + *Assignment* + *umap* + *Gabor*
Madelon	74.6	51.9	50.0	63.9	64.6	79.6	**86.2**	*tsne (hamming)* + *Blurring* + *umap* + *Gabor*
Ringnorm	95.7	73.9	97.6	98.0	**98.2**	96.4	96.5	*tsne (hamming)* + *Blurring* + *umap*
*Average*	81.8	78.1	75.8	74.9	84.0	86.4	**88.7**	

The highest values are highlighted in bold text.

On the scRNA-seq + ATAC-seq dataset, MRep-DeepInsight achieved a classification accuracy of 90.5% on the test set, surpassing random forest and DeepInsight by over 7% and 2.3%, respectively. It also exceeded the performances of XGBoost, LightGBM, and FT-Transformer by 11.5%, 15.2%, and 1.8%, respectively. For the scRNA-seq dataset, MRep-DeepInsight demonstrated improved performance, reaching 90.0%. The second-best result was obtained by DeepInsight at 89.2%, followed by L2-regularized logistic regression at 87.7% and FT-Transformer at 86.2%. In the case of the ATAC-seq only dataset, MRep-DeepInsight achieved an accuracy of 79.4%, slightly ahead of L2-regularized logistic regression, which posted 79.0%.

When applied to the Alzheimer's disease dataset, the FT-Transformer achieved the highest results with an accuracy of 90.1%, followed by MRep-DeepInsight at 89.3%. MRep-DeepInsight demonstrated improvements in accuracy on the Madelon dataset, reaching 86.2%, compared to 79.6% by the DeepInsight method. Additionally, for the Ringnorm dataset, the FT-Transformer recorded the highest accuracy at 98.2%, closely followed by LightGBM at 98.0%. It can be observed that the Alzheimer dataset, which has the smallest number of samples, and the Ringnorm dataset, which has the fewest number of features, both showed superior performance with the FT-Transformer, indicating its effectiveness in handling datasets with limited size and complexity.

Across all six datasets, MRep-DeepInsight achieved an average classification accuracy of 88.7%, which is 6.9% higher than that of random forest, 10.6% higher than L2-regularized logistic regression, and 2.3% higher than the DeepInsight method. Furthermore, the FT-Transformer recorded the highest accuracies on the Alzheimer and Ringnorm datasets, with an overall average of 84.0% across the six datasets. The average accuracies for XGBoost and LightGBM were 75.8% and 74.9%, respectively. These results highlight the utility of the MRep-DeepInsight approach in handling diverse datasets. Although the users may be recommended to use FT-Transformer when the datasets are limited in size and complexity as shown above.

### Dependency on models and parameter values

This subsection examines the dependencies in the obtained results by conducting an comparitive study. This study involves varying parameters, including changing the CNN architecture from ResNet-50 to EfficientNet-B6. For ResNet-50, the hyperparameters are tuned using the Bayesian optimization technique whereas the following hyperparameters (learning rate = 0.001, weight decay = 0.0001 and momentum = 0.9) were used for EfficientNet-B6. The mini-batch size and epochs are adjusted based on the memory capacity of the GPU used. The results obtained using EfficientNet-B6 are presented in Supplement Figure S2. Additionally, the effects of varying representation schemes are illustrated in Supplement Table S7 (see Sect. 5 of Supplement File 1 for more discussion). This evaluation provides insights into the impact of different parameters and representation schemes on the performance of the MRep-DeepInsight methodology, thereby helping to assess its robustness and generalizability.

## Discussions

The MRep-DeepInsight methodology presented offers a novel approach to tabular data analysis by leveraging the power of CNNs and multiple image representations. Our findings demonstrate that MRep-DeepInsight outperforms several state-of-the-art classifiers, including random forest, L2-regularized logistic regression, XGBoost, LightGBM and FT-Transformer and the original DeepInsight method on datasets tested during this study. There is a possibility of improving the classification performance further by incorporating advanced CNN architectures such as Noisy-Student EfficientNets^25^, increasing the number of channels of layers to reduce the overlapping of elements^26^, or through employing mutual information of elements^46^.

One of the key strengths of MRep-DeepInsight lies in its ability to generate multiple representations of non-image data, thereby enriching the information available. By converting tabular data into images using manifold techniques and subsequently utilizing CNN architectures, MRep-DeepInsight captures intricate patterns and fine-grained details that are often overlooked by traditional machine learning methods. This capability can be particularly useful in complex domains such as medical research, where accurately identifying phenotypes and diagnosing diseases is of paramount importance.

Our experimental results demonstrate the effectiveness of MRep-DeepInsight on six diverse datasets. Notably, MRep-DeepInsight achieved improvements in classification accuracy compared to other ML methods. The results confirm the ability of the framework to leverage the strengths of gene expression data for improved classification within appropriate context. Similar trends were observed on other datasets, where MRep-DeepInsight consistently surpassed or matched the performance of existing methods.

The average improvement of 2.3% in classification performance, especially the 7.3% enhancement over random forests, is substantial in fields where even minor accuracy gains are very important. The FT-Transformer and LightGBM also produced very high classification accuracies. Nonetheless, MRep-DeepInsight was able to deliver more than 2% improvement compared to these sophisticated methods. Such improvements are noteworthy, as evidenced by the attention given to advancements in the field, such as the 0.2% improvement achieved by Xie et al.^25^ with their Noisy-Student enhancement over Big Transfer (BiT-L). This underscores the importance and impact of even modest performance gains in the highly competitive fields of machine learning and deep learning. The improvement of 2.8% in classification accuracy over DeepInsight, while seemingly modest, can be of practical value in the fields such as biomedical research and precision medicine, where improved accuracy can lead to better diagnostic and predictive outcomes. For example, in the Alzheimer’s disease dataset, an 2.3% improvement can impact early diagnosis and treatment strategies Similarly, in the Madelon dataset, a 6.6% increase in accuracy can enhance the reliability of predictions in this artificial dataset.

The improved performance of MRep-DeepInsight over DeepInsight when applied to the Madelon dataset can be attributed to the complexity and artificial nature of the dataset. Madelon is a synthetic dataset that was specifically created for the NIPS 2003 feature selection challenge, and it is known to be particularly challenging due to its properties such as redundant and irrelevant features and non-linearity. MRep-DeepInsight offers an advantage in this context by leveraging multiple representations of the dataset to enhance the spatial information available for the CNN. The approach is effective by increased spatial information, diverse representations, and enhanced feature selection. Therefore, it formats the dataset more effectively for CNN, thereby improving the classification performance over DeepInsight approach. Conversely, on the ATAC dataset, which is characterized by sparse features, establishing spatial relationships is more challenging. In such scenarios, methods like FT-Transformer have shown improved performance (80.1%) compared to MRep-DeepInsight (79.4%), as observed from the results.

We have also analyzed a case where traditional data augmentation techniques were applied to images transformed by DeepInsight. While this method may enhance variability within the training set, it could distort the spatial relationships that DeepInsight aims to preserve. For example, reflection could create mirror images that no longer correspond to the original spatial correlations of features. Similarly, translation and scaling might move features away from their original pixel locations, potentially disrupting the structural patterns expected by the CNN for the test set.

Our analysis revealed that applying traditional data augmentation techniques to DeepInsight resulted in a loss of test accuracy and an increase in training time compared to multi-representation, indicating it is not suitable for this context. Nonetheless, running the training process for a very long time could reduce this risk. In contrast, the MRep-DeepInsight approach, by generating multiple manifold representations, could offer a more nuanced and effective way to introduce variability and robustness while preserving the spatial coherence important for CNN performance on the test set. These findings are elaborated in Supplement File, Sect. 9.

Furthermore, this improvement demonstrates the effectiveness of our approach in complementing the strengths of CNNs for structured data analysis, extending its applicability to Vision Transformer (ViT) domains as shown by Gokhale et al.^26^. The efficacy of the DeepInsight framework, along with its variants, has been independently validated across diverse datasets, showcasing competitive performance against state-of-the-art machine learning methods like XGBoost.

For instance, Neto et al.^47^ illustrated that a convolutional layer with DeepInsight could match the performance of XGBoost on Arboviruses data. Similarly, Khan et al.^48^, employed DeepInsight with ResNet-50 on a Truck-Involved Crash Dataset, achieving superior results over XGBoost and other methods. Moreover, Rahim and Hassan^18^ utilized it for traffic crash severity prediction, and Ravaee et al.^46^ demonstrated their IP3G model, based on DeepInsight, in detecting phenotypes in an unsupervised manner, surpassing traditional clustering methods. Akkaya and Kalkan^49^ applied a DeepInsight variant in their One2MFusion method, showing superior performance on Alzheimer’s disease study datasets from the NCBI database. Andresini et al.^31^ applied its variant, MAGNETO, for intrusion detection, showcasing competitive performance. Tran et al.^19^, also developed DeepInsight for intrusion detection system. Jiang and Liao^50^ used DeepInsight for credit card fraud detection, and Gokhale et al.^26^ integrated it with ViT across ten gene expression datasets, showing superior performance over competing methods, like XGBoost and 1D-CNN. Kanber et al.^13^, applied DeepInsight to the sparse MNIST database, and Pasquadibisceglie et al.^15^, incorporated it within their ORANGE method for outcome predictions on diverse event logs. Tajmirriahi et al.^17^, also drew inspiration from DeepInsight for P300 detection in time-series EEG signal analysis.

In summary, these examples illustrate the broad applicability of DeepInsight beyond simple classification tasks. It has been effectively utilized for feature selection^27,28^, integrated with methods like XGBoost and Random Forest^29,30^, and ViTs^26^, employed in identifying gene subsets for pathway analysis^8^, and applied for multi-omics data analysis^7^. Additionally, DeepInsight has been used in generating data through GANs^30,31^, and processing multi-omics data across multiple layers^7^. These varied uses of DeepInsight also highlights the potential of incorporating MRep-DeepInsight, not just as a classifier, but could be used as a comprehensive tool for data analysis and interpretation tasks.

Furthermore, the utilization of well-established CNN architectures, specifically ResNet-50^32^ and EfficientNet-B6^33^, contributed to the success of MRep-DeepInsight. Both ResNet-50 and EfficientNet-B6 have been pre-trained on extensive datasets like ImageNet. This pre-training enables the models to benefit from transfer learning, where knowledge from one domain (image recognition) is carried over and applied to another (specific tabular data converted to image form). This is advantageous for feature extraction where pre-trained models can detect nuanced patterns that may not be immediately apparent in smaller or domain-specific datasets. The integration of transfer learning significantly enhanced the feature extraction process leading to improved classification results.

It is worth noting that the performance of MRep-DeepInsight was evaluated using rigorous experimental setups, including training/validation/test set divisions and hyperparameter optimization. The use of separate test sets ensured an unbiased assessment of the final model's performance. The hyperparameter optimization process, including grid search optimization and Bayesian optimization, allowed us to fine-tune the models and achieve optimal results.

Overall, the results obtained from our experiments highlight the effectiveness and potential of the MRep-DeepInsight methodology for tabular data analysis. By integrating multiple image representations and leveraging CNN architectures, MRep-DeepInsight can be a robust and accurate approach for solving various classification tasks. The improvements in classification accuracy observed across tested datasets suggest possible utility of MRep-DeepInsight in diverse domains, including single-cell analysis, Alzheimer's disease diagnosis, and artificial datasets.

While this study presents some evidence for the superiority of MRep-DeepInsight, further research is needed to explore its applicability in other domains and to investigate its performance on larger and more diverse datasets. Additionally, future work could focus on optimizing the methodology for efficiency and scalability, as well as exploring potential extensions or adaptations of the approach for other types of data.

Beyond the measured increase in accuracy, the method's integration of multiple manifold techniques offers a representation of data that captures a broader spectrum of its inherent structure. This multifaceted approach enables a deeper understanding of complex data relationships and patterns that might be overlooked by single representation methods. It fosters better generalization, interpretability, and robustness, making it a tool that can be fine-tuned for diverse research requirements and data types.

In summary, MRep-DeepInsight offers a novel strategy for tabular data analysis by harnessing the power of multiple image representations and CNN architectures. We believe that the proposed methodology makes an important contribution to this field as it is broadly applicable in a variety of different contexts.

## Conclusion

In conclusion, the Multi-representation DeepInsight (MRep-DeepInsight) methodology presented in this study offers a novel and effective approach to tabular data analysis. By generating multiple representations of non-image data and utilizing CNNs, MRep-DeepInsight achieves demonstrable improvements in classification accuracy and can provide valuable insights into the underlying data structure. The integration of manifold techniques and the use of well-established pre-trained CNN architectures, namely ResNet-50 and EfficientNet-B6, contribute to the enhanced performance of MRep-DeepInsight.

The experimental results across various datasets demonstrate better performance of MRep-DeepInsight over state-of-the-art classifiers on a diverse selection of application cases. The methodology's ability to convert tabular data into images and leverage CNNs for classification potentially allows more accurate identification of phenotypes and improved disease diagnosis. The findings will be of particular relevance for single-cell analysis, medical research, and artificial datasets. While MRep-DeepInsight shows improvements, there is still room for further exploration and optimization. Future research can focus on scalability, adapting the methodology to different data types, and comparing it with other advanced techniques to unlock its full potential in real-world applications.

Overall, the MRep-DeepInsight methodology is a promising strategy for tabular data analysis that can enhance classification accuracy and offer valuable insights into complex datasets. Its versatility and performance improvements make it a valuable tool for researchers seeking to uncover hidden patterns and gain deeper insights from their data, driving advancements in various research domains.

## Methods

In the DeepInsight framework, elements of feature vectors are spatially mapped, creating relationships that, in the original version, are constrained to a single representation. MRep-DeepInsight diversifies this by employing multiple manifold techniques, allowing for a more comprehensive and dynamic relationship between these elements.

For instance, considering two elements, $g_{i}$ and $g_{j}$, within a $d$-dimensional feature vector in the DeepInsight framework, where,

$$x = [g_{1} ,g_{2} ,...,g_{i} ,...g_{j} ,...g_{d} ]^{T} \in \Re^{d}.$$

The relationship between these two elements, in terms of distance within the pixel-framework, is depicted as, $\left| {\left| {L\left( {g_{i} } \right) - L\left( {g_{j} } \right)} \right|} \right|$, where $L\left( \cdot \right)$ denotes the location in terms of rows and columns in the local pixel region, $W$ (receptive field).

In the original DeepInsight framework, only one manifold method is used to generate points in the pixel-framework, limiting the relationship between these points to one unique representation. This relationship can be simplified as,$D = \left| {\left| {L\left( {g_{i} } \right) - L\left( {g_{j} } \right)} \right|} \right|$, given the receptive field $W.$

This influences the weights and biases of CNN architecture during the training phase. Therefore, any element $g$ represented in the DeepInsight framework can be depicted as $L\left( g \right)$.

In CNN, data augmentation via rotation, translation, reflection or random scaling helps to address issues like overfitting and data scarcity, and it makes the model robust with better performance. However, in the DeepInsight framework, due to the fixed relationship between $g_{i}$ and $g_{j}$, this is not possible or rather limited (although averaging of two transformed image samples are possible). Therefore, the fixed positioning may impact robustness adversely especially when the training samples are not sufficiently large.

MRep-DeepInsight extends DeepInsight by introducing flexibility through the employment of multiple manifold techniques which provides a comprehensive relationship between, $g_{i}$ and $g_{j}$. This is expressed as

$$D_{r} = ||L(g_{i} |\lambda_{r} ) - L\left( {g_{j} |\lambda_{r} } \right)||$$

In a given receptive field $W$, where $\lambda_{r}$ is the $r$-th manifold technique and $r = 1,2,...,m$.

Therefore, in MRep-DeepInsight, the representation of any element $g$ can be expressed as $L(g|\lambda_{r} )$ with integrated representation given as $\cup_{r = 1}^{m} L(g|\lambda_{r} )$. This approach theoretically allows modeling of any function with an infinite number of mapping techniques, i.e., $\cup_{r = 1}^{\infty } L(g|\lambda_{r} )$.

The additional flexibility offered by MRep-DeepInsight enhances the robustness of model estimation. The extended distance measure now includes $m$ possibilities compared to the original single representation, necessitating a revised approach for class label evaluation in its accordance.

This section introduces the MRep-DeepInsight methodology proposed in this study. The model comprises three key components: 1) the transformation of images using multiple DeepInsight representations, 2) the utilization of ResNet-50 and EfficientNet-B6 models as the CNN architecture, and 3) the classification of test samples through average weighting (see Fig. 1). The following subsections outline the step-by-step procedures involved in the MRep-DeepInsight methodology.

### Transformation phase of MRep-DeepInsight

This phase allows the conversion of tabular data to multiple representations of image samples for CNNs in an unsupervised manner. Let us consider a sample represented by **x**_*j*_, where **x** has $d$ elements or features (rows), and $j$ denotes the samples (columns); i.e.,

$$x_{j} = [g_{1j} ,g_{2j} ,...,g_{dj} ]^{T}$$

Therefore, the training data can be represented as $M = \left\{ {x_{j} } \right\}$ for $j = 1,2,...,n$, where $n$ is the number of samples. Consequently, the tabular data during the training phase is denoted as $M \in \Re^{d \times n}$. The DeepInsight model^6^ is utilized to convert the non-image data $M$ into image data $E = \left\{ {e_{j} } \right\}$ for $j= 1,2,...,n$. Each image sample, $e_{j}$ has a size of $p \times q$. The DeepInsight transformation incorporates manifold techniques such as t-SNE^11^ using different distances (e.g., cosine, Euclidean, Mahalanobis and Chebychev), UMAP^12^, Kernel PCA^51^ and PCA^52^ (see Sect. 6 of Supplement File 1 for more details about these techniques). These manifold mappings can be further enhanced by applying blurring techniques^23^, Gabor filtering^24^ and the assignment distribution algorithm^53^. The DeepInsight pipeline incorporates several additional steps to enhance the transformation process. These steps include the implementation of the convex hull algorithm, rotation of Cartesian coordinates, determination of pixel locations, and mapping of elements to their respective pixel locations (more discussions on feature mapping are given in Sect. 3 of Supplement File 1).

When using t-SNE, the algorithm constructs a probability distribution that captures the similarity between pairs of samples. Samples with a higher degree of similarity are assigned higher probabilities, while dissimilar samples receive lower probabilities. This probability distribution is then projected onto a two-dimensional plane, allowing for visualization and analysis. To align the distributions, the Kullback–Leibler divergence is minimized, ensuring that the transformed representations accurately represent the relationships and structure within the data.

It is important to note that these manifold techniques are not only employed for sample visualization, but also for visualization of the genes or elements. To achieve this, the transpose of data $M$ is used to determine the pixel locations, $P$. Many of these techniques can project data onto a 2D plane. Thus, if $d > 2$ and $n > 2$, it becomes possible to obtain a 2D framework for $M$. The pixel locations can be obtained using the equation:

1 $$P = H\left( M \right)$$

Here, $H$ denotes the DeepInsight transform for finding pixel locations, and $M$ corresponds to a layer of the training set (e.g. gene expression data). Note that the transpose operation in Eq. (1) is not explicitly shown. Once the framework of the pixel locations is determined using Eq. (1), the elements can be mapped to generate the corresponding images:

2 $$e_{j} = \Phi \left( {x_{j} } \right)\;{\text{for}}\;j = 1,2,...,n$$

Each non-image sample, $x \in \Re^{d}$, is mapped to an image sample, $e_{j} \in F^{p \times q}$, where $F$ is a pixel-coordinates system, $\Phi$ is a transformation function ($\Phi :x \to e$), whereas $p$ and $q$ denote the number of rows and columns, respectively. The transformation, $\Phi$, provides location information for $i$-th element, $g_{ij} \forall j = 1,2...n$. If the $i$-th location is depicted by $\left[ {a_{i} ,b_{i} } \right]$, where, $a_{i}$ is the $i$-th row and $b_{i}$ is the $i$-th column of the pixel-coordinates, $F$, then $g_{ij}$ will be mapped at this location, $\left[ {a_{i} ,b_{i} } \right]$, with its corresponding value.

Different manifold schemes will provide a different $\Phi$ transformation. If $\Phi_{r}$ denotes the $r$-th representative mapping, then $\Phi_{r} :x \to e^{\left( r \right)}$ for $r = 1,2,...,m$, i.e., $m$ mappings for a sample will be obtained. Mapping all the elements $\left( {i = 1,2,...,d} \right)$ corresponding to $j$-th sample, $x_{j}$, would produce an image sample, $e_{j}^{\left( r \right)}$. Therefore, we can generalize Eq. (2) as:

3 $$e_{j}^{\left( r \right)} = \Phi_{r} \left( {x_{j} } \right)\;{\text{for}}\;j = 1,2,...,n$$

This will, in turn, create $r$ locations for the *i*-th element; i.e., $\Phi_{r}$ contains $\left[ {a_{i}^{\left( r \right)} ,b_{i}^{\left( r \right)} } \right]$ location information for $i = 1,2,...,d$.

The transformation process also includes normalization of the values, typically between the range of [0,1] or [0,255]. In this work, we employed the norm-2 normalization, which was introduced in DeepInsight^6^.

Based on Eq. (2), the layer of image data obtained from the transformation is represented as:

4 $$E = \left\{ {e_{1} ,e_{2} ,...,e_{n} } \right\}$$

Equation (4) produces one representation ($r = 1$) specific to a manifold method used. For multiple representations $(r > 1)$, Eq. (4) can be rewritten as:

5 $$E_{r} = \left\{ {e_{1}^{\left( r \right)} ,e_{2}^{\left( r \right)} ,...,e_{n}^{\left( r \right)} } \right\},\;{\text{for}}\;r = 1,2,...,m$$

where, $E_{r} \in F^{p \times q \times n}$. Here, we obtain $m$ representations of the image sample, $e_{j}^{\left( r \right)}$, corresponding to a given sample, $x_{j}$. Hence, by creating $m$ representations for each sample, the total number of image samples becomes $nm$.

In this study, a total of $m = 70$ representations can be generated. For more details, see Supplement Figure S1, Supplement Table S1 and Sect. 1 of Supplement File 1 titled “Multiple representations to find the characteristics of a sample”.

### Model estimation for classification

**Figure 2:**
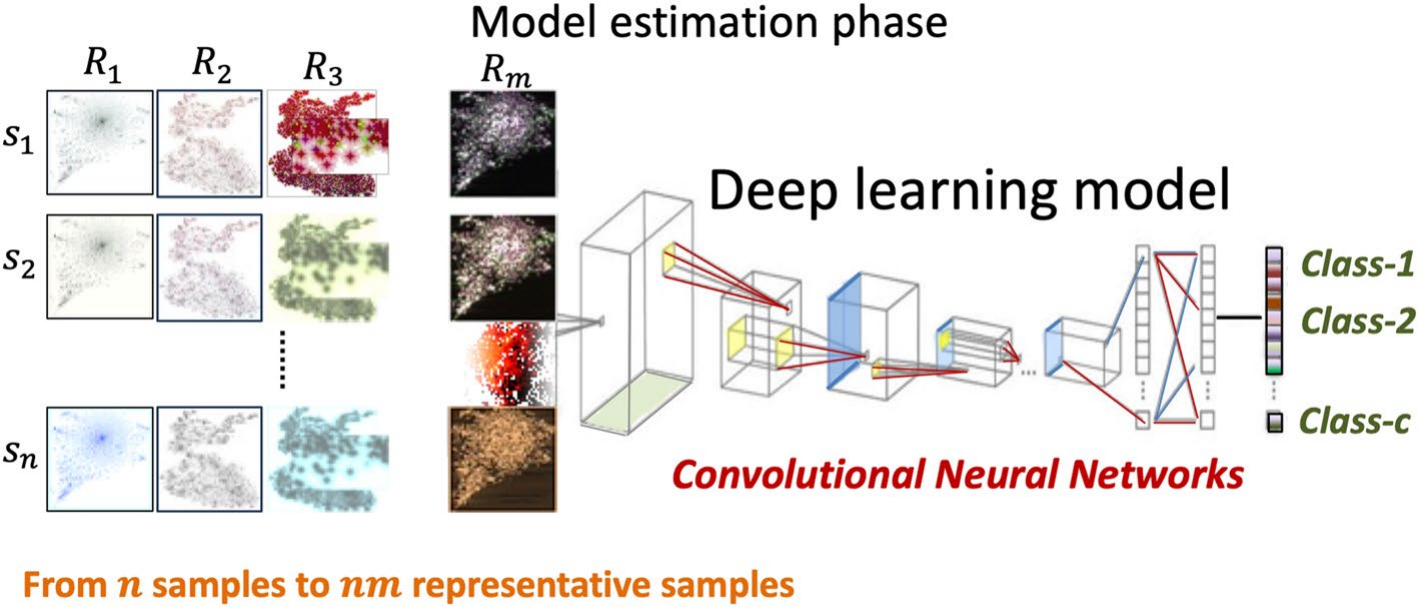
Image samples converted by MRep-DeepInsight are processed by deep learning model.

In this paper, we focus on the classification task using CNN architectures. However, the DeepInsight framework can be utilized for other architectures and tasks^8,26,30,31^. Here, two well-established CNN models, namely ResNet-50 and EfficientNet-B6, are employed to perform accurate and robust classification of the generated image representations. Figure 2 depicts deep learning model is processing the image samples converted by MRep-DeepInsight.

ResNet-50 is a deep CNN architecture renowned for its ability to effectively address the challenges associated with training deep networks. With its residual connections, ResNet-50 enables the successful training of models consisting of 50 layers, facilitating the extraction of intricate features from the input images. This architecture has demonstrated exceptional performance across various image analysis tasks, making it a suitable choice for the classification component of MRep-DeepInsight. ResNet-50 leverages the power of transfer learning as it is pre-trained with large image datasets, enhancing its feature extraction capabilities and contributing to its robustness and effectiveness^32^.

EfficientNet-B6^33^, on the other hand, is part of the EfficientNet family of CNN architectures known for their superior efficiency and performance. These models achieve a remarkable balance between accuracy and computational efficiency by utilizing a compound scaling technique that optimizes the depth, width, and resolution of the network. EfficientNet-B6 specifically offers a powerful capacity to capture fine-grained details and intricate patterns within image representations. Like ResNet-50, EfficientNet-B6 also benefit from transfer learning, having been pre-trained with large image datasets, which significantly enhances its feature extraction capabilities and adds to the overall robustness and effectiveness of MRep-DeepInsight.

By leveraging the capabilities of both ResNet-50 and EfficientNet-B6, the MRep-DeepInsight methodology benefits from the rich hierarchical feature extraction abilities of these CNN architectures, enabling accurate classification of the multiple image representations and providing valuable insights into the underlying structure and characteristics of the data.

Both ResNet-50 and EfficientNet-B6 are supervised models, requiring the inclusion of target or class label information along with samples. To define explicitly, let $X = \left\{ {x_{j} } \right\}$ for $j = 1,2,...,n$ represent the tabular data with $n$ training samples with $d$ elements (or features). Additionally, let $\Omega = \left\{ {\omega_{j} } \right\}$ be the corresponding class labels, where $\omega = \left\{ {1,2,...,c} \right\}$ and $c$ is the number of classes. The tabular data $X$ is converted into images using Eq. (5), resulting in $m$ sets, $E = \{ E_{1} ,E_{2} ,...,E_{m} \}$. This procedure generates $nm$ images for the $n$ training samples, which are then inputted into the CNN architecture for training.

If we define a CNN architecture with its various layers as $\Psi$, then its output can be depicted as:

6 $$W = \Psi \left( {E,\Omega ,\theta ,W} \right)$$

Here, $E$ depicts the training set consisting of $nm$ images, $\Omega$ denotes the class labels of all the samples, $\theta$ encompasses the set of all hyperparameters, and $W$ represents the weights and biases of the pre-trained CNN model. After training $\Psi$ over several epochs, we obtain updated weights and biases, $W$. This updated model is then utilized for the classification of new samples, enabling accurate predictions based on the learned patterns and representations.

### Model analysis phase

**Figure 3:**
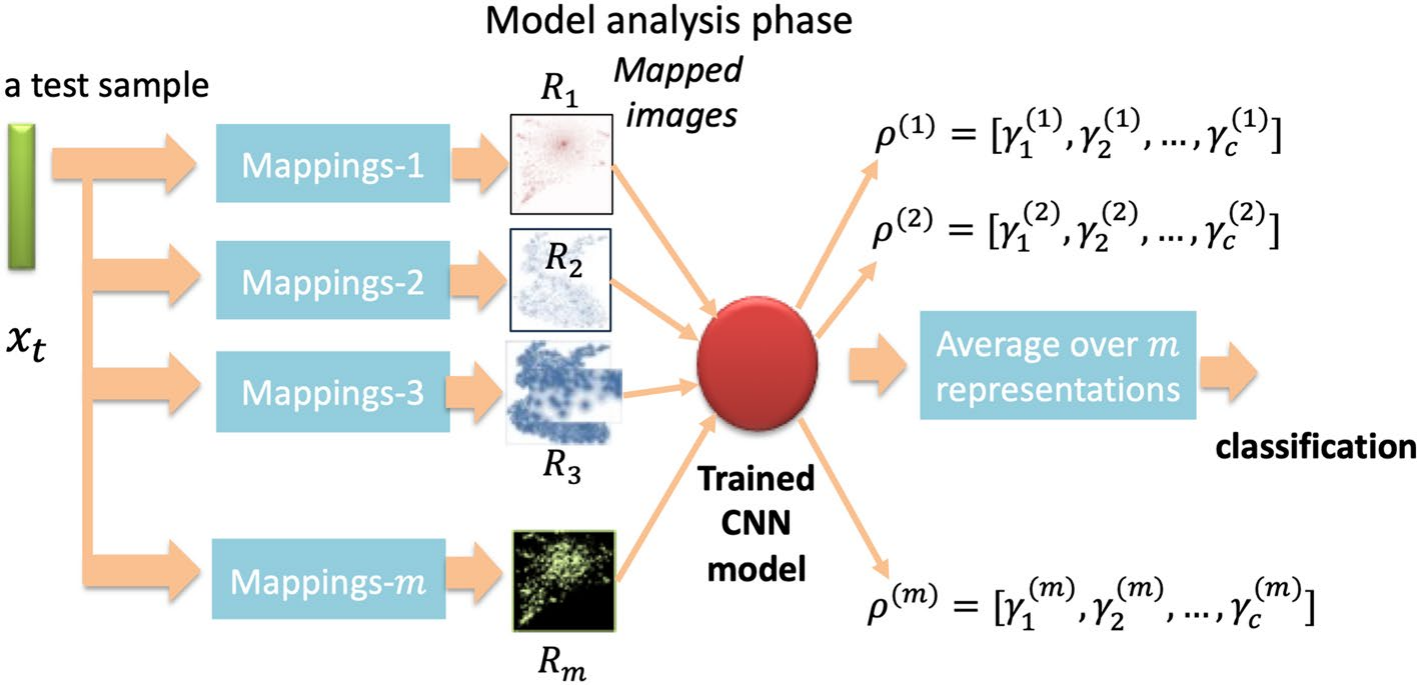
Model analysis phase: a novel test sample is analyzed to one of the defined classes.

In the model analysis phase (Fig. 3 depicts an overview of the model analysis phase), a sample is processed through the trained CNN model (Eq. (6)) to obtain predicted probabilities, which are then used to determine its categorization into one of $c$ classes. Let us consider an independent test sample, $x_{t}$, that was not previously used during the training phase. It is first converted into $m$ multiple representations using location information from Eq. (3), resulting in $m$ images corresponding to $x_{t} :$

$$Ts = \Phi_{r} \left( {x_{t} } \right)$$

Here $Ts \in F^{p \times q \times m}$ represents the set of $m$ mapped images $Ts = \left\{ {R_{1} ,R_{2} ,...,R_{m} } \right\}$, where each image is denoted as $R_{r} \in F^{p \times q}$. These $m$ images are then fed into the trained CNN model, and each image $R_{r}$ retrieves a probability distribution from the model:

7 $$\rho^{\left( r \right)} = \psi \left( {W,R_{r} } \right)\;{\text{for}}\;r = 1,2,...,m$$

where $\psi$ provides $c$ probabilities for the image $R_{r}$ from the trained CNN model, represented as $\rho^{\left( r \right)} = \left[ {\gamma_{1}^{\left( r \right)} ,\gamma_{2}^{\left( r \right)} ,...,\gamma_{c}^{\left( r \right)} } \right]$ or ${\rho_{j}^{\left( r \right)}}$. In order to classify the sample, $t$, we can calculate the average probability from Eq. (7) to determine the class label:

8 $$\rho_{avg} = \frac{1}{m}\mathop \sum \limits_{r = 1}^{m} \rho_{}^{\left( r \right)} = \mathop \sum \limits_{r = 1}^{m} \left[ {\gamma_{1}^{\left( r \right)} ,\gamma_{2}^{\left( r \right)} ,...,\gamma_{c}^{\left( r \right)} } \right]$$

The class label can now be determined from Eq. (8) as:

9 $$\omega = {\text{arg}}\mathop {{\text{max}}}\limits_{{\phantom{0}}} \rho_{{{\text{avg}}}} = {\text{arg}}\mathop {{\text{max}}}\limits_{{\phantom{0}}} \left( {\mathop \sum \limits_{r = 1}^{m} \rho_{\phantom{0}}^{\left( r \right)} } \right)$$

where $\omega$ in Eq. (9) depicts the class label assigned to the sample, $x_{t}$, based on the highest average probability. This completes the procedure, providing the classification of the test sample using the MRep-DeepInsight methodology.

It should be noted that in the case of DeepInsight framework, a novel test sample, $x_{t}$, was labelled using:

$\omega = {\text{arg max}} \left( {\gamma_{j} } \right)$, where $\gamma_{j}$ for $j = 1,2,...,c$ is the probabilities estimated by the $c$-class model.

However, in the MRep-DeepInsight framework, since the positions between the two elements augmented by the integration of manifold method, $\lambda_{r}$, for $r = 1,2,...,m$, this increases the probabilities in a similar order, i.e.,

$\rho_{j} |\lambda_{r}$ or depicted here as $\rho_{j}^{\left( r \right)}$, where $j = 1,2,...,c$ and $r = 1,2,...,m$

Thus, class label estimated for a test sample $x_{t}$ is given by Eq. (9).

## Supplementary Information

Supplementary Information.

## Data Availability

The single cell data can be downloaded from the link https://support.10xgenomics.com/single-cell-multiome-atac-gex/datasets/1.0.0/pbmc_granulocyte_sorted_10k. Cell Ranger ARC pipeline, which includes counting UMIs based on the fastq data is available at https://support.10xgenomics.com/single-cell-multiome-atac-gex/software/pipelines/latest/what-is-cell-ranger-arc. The Alzheimer’s disease (AD) dataset is available from the Gene Expression Omnibus (GEO) database at the National Center for Biotechnology Information (NCBI) (https://www.ncbi.nlm.nih.gov/geo/). The exact configuration of AD data is also available at GitHub link https://github.com/alok-ai-lab/MRep-DeepInsight/blob/main/Data/dataset4.mat. Madelon dataset is available from UCI repository https://archive.ics.uci.edu/dataset/171/madelon and Ringnorm dataset is available from the University of Toronto repository https://www.cs.toronto.edu/~delve/data/ringnorm/desc.html.

## Code availability

MRep-DeepInsight is available as a MATLAB package, the latest version can be downloaded from the link https://github.com/alok-ai-lab/MRep-DeepInsight. The ReadMe file with examples is also given to guide a user to execute the codes.
